# Exposure to violence and associated factors among university students in Ethiopia: A cross-sectional study

**DOI:** 10.1371/journal.pone.0319792

**Published:** 2025-03-18

**Authors:** Wudinesh Belete Belihu, Tobias Herder, Minilik Demissie Amogne, Jesper Sundewall, Jack Palmieri, Anette Agardh

**Affiliations:** 1 Social Medicine and Global Health, Malmö, Sweden; 2 Infectious Disease Research Directorate, Ethiopian Public Health Institute, Addis Ababa, Ethiopia; IFPRI: International Food Policy Research Institute, UNITED STATES OF AMERICA

## Abstract

**Background:**

Violence is a major public health concern with a significant impact on the health and well-being of individuals, families, and communities. Living in a new environment without parental control and experimenting with new lifestyles may increase the risk of violence among university students. Therefore, this study aimed to assess exposure to violence and its associated factors among university students in Ethiopia.

**Method:**

A cross-sectional study was conducted among 2988 university students from six randomly selected universities in Ethiopia. A two-stage stratified sampling method was used to recruit the study participants. A self-administered questionnaire was utilized to collect information regarding exposure to emotional, physical, and sexual violence. Bivariable and multivariable logistic regression analyses were used to identify factors associated with violence exposure in the last 12 months.

**Results:**

The prevalence of exposure to any type of violence in the last 12 months was 17.6% (n = 525) (17.9% among males, 16.5% among females). The adjusted odds ratio (AOR) of violence was 2.9 times higher (95% CI 1.6-5.0) among students older than 25 years than those aged 18-20 years. Those students who were in a relationship had 1.4 times higher odds of violence (95% CI 1.0-2.0) than those who were not in a relationship. In addition, those students who were from rural residences before coming to the university had 1.4 times higher odds of violence (95% CI 1.1-1.8) than those from urban residences. The odds of violence among those who consumed alcohol once a week or more in the past month were 2.2 times higher (95% CI 1.3-3.6) than those who did not consume alcohol. Furthermore, the likelihood of violence was 1.6 times higher (95% CI 1.0-2.4) among those who chewed khat and 2 times higher (95% CI 1.3-3.1) among those who used other drugs in the last 12 months.

**Conclusion:**

Exposure to violence is a challenge for both male and female university students in Ethiopia. Several socio-demographic and behavioral factors were significantly associated with exposure to violence. Therefore, it is crucial for universities and stakeholders to raise awareness about contributing factors to minimize violence, regardless of gender.

## Background

Globally, violence remains a pervasive issue, manifesting in forms as varied as armed conflict, terrorism, domestic violence, and crime, impacting millions of lives and destabilizing societies. Both men and women across the world are victims of violence. For example, according to the estimates based on the WHO global database from 161 countries on the prevalence of violence against women, more than one in four (27%) ever-partnered women aged 15–49 years has experienced physical and/or sexual intimate partner violence since the age of 15 years and one in seven (13%) experienced this violence in the past year before the survey [1].

Violence is a public health issue due to its widespread prevalence and adverse short and long-term impacts on physical, mental, sexual, and reproductive health. Additionally, it entails significant social and economic costs for victims, their families, and societies, including intergenerational cycles of violence, lower academic performance and reduced productivity [2–4]. Violence is frequently categorized into four types, physical, sexual, psychological violence, and violence involving deprivation or neglect [5]. According to the 2016 Ethiopian Demographic and Health Survey (EDHS), among women aged 15-49, the prevalence of physical violence ever and in the past 12 months was 23% and 15%, respectively; the prevalence of sexual violence ever and in the past 12 months was 10% and 7%, respectively [6]. Although both men and women are victims of violence, limited information is available concerning men’s exposure to violence in Ethiopia or neighboring countries.

University students can be particularly at risk for exposure to violence. For many young adults, university study is the first time that they are away from parental control, which can increase their exposure to behaviors associated with experiences of violence, such as alcohol consumption [7–9] and drug use [10–12]. Furthermore, university is a time of self-discovery and exploration of new things, such as being in relationships and having sex. While students are often independent and make their own decisions, they are also subject to power imbalances by peers and teachers, which might increase their exposure to violence. It is difficult to get a clear picture of campus violence due to underreporting of incidents and the use of inconsistent survey methods across studies [13]. Exposure to emotional and physical violence in the past six months among university students in the US and Canada was 17% and 16% among males and females, respectively [14]. Another study among university students in Italy showed that experience of psychological and physical violence among peers/at school was higher among males than females (21.5% vs. 9.7%) [15]. Exposure to violence is also prevalent among university students in African countries. A study on sexual coercion, interpersonal violence, and mental health among university students in southwestern Uganda showed that 31.1% of the respondents had experienced some form of sexual coercion in their lifetime; 27.8% and 9.6% of the respondents had been exposed to perceived threats/threats of violence and actual physical violence, respectively, during the past year [16]. Furthermore, a study on the prevalence of sexual violence among female university students in Ethiopia showed that 9.8% and 1.6% of students had been victims of completed rape in their lifetime and after joining the university, respectively [17]. A similar study among female university students in Ethiopia revealed a 15.3% lifetime prevalence of rape and a prevalence of 8% and 2.3% since joining the university and in the current academic year, respectively [18].

### Theoretical framework

It has been argued that a combination of individual, institutional, community, public policy and societal factors, as well as group processes contribute to exposure to violence in higher education across countries [13]. One way of understanding these vulnerabilities further would be to view them from the perspectives of developmental ecology. According to Bronfenbrenner’s ecological model of human development, youth violent behavior should be viewed as a result of interactions within various environmental systems rather than in isolation [19]. The model comprises five subsystems, the micro-, meso-, exo-, macro- and chronosystems. The microsystem includes immediate relationships like family, school/university, and peers, the mesosystem includes interactions between various aspects of the microsystem, and the exosystem encompasses external influences such as policies and institutions that indirectly affect individuals. The lack of curriculum and policy to prevent or manage exposure to violence, is a general problem in Ethiopia, not least for university students, and can be seen as part of the exosystem in the human deveopment model. Furthermore, university students may be influenced by the macrosystem, which involves broader cultural beliefs and ideologies and the chronosystem, accounting for changes over time [20].

Factors such as individual characteristics (age, gender) and behaviors (substance use) interact with these systems to increase vulnerability to violence among youth, particularly among university students who may face unique risks due to their social networks and environmental contexts. A study by Heise of violence among women that adopted an integrated ecological framework, supported by findings from international and cross-cultural research, described factors contributing to violence at different levels of the social ecology [21]. These factors involve determinatnats at the individual level, such as having witnessed marital violence as a child and having been abused as a child, the microsystem level, including male dominance in the family, the exosystem level, including low socio-economic status and delinquent peer association, and the macrosystem level, including masculinity norms linked to aggression and dominance, rigid gender roles, and acceptance of interpersonal violence [21]. Another longitudinal study of the development of serious delinquent behavior among adolescent boys showed the relationships between microsystem influences of parenting and peer deviance, macrosystem influences of community structural characteristics and neighborhood social organization, and individual involvement in violence [22]. Thus, interpersonal violence may have multiple determinants existing on interacting levels.

Previous studies conducted in Ethiopia, South Africa, India, and Turkey showed that factors significantly associated with violence exposure among university students were age [23], childhood rural residence [24], being a second-year student [25], being married or living with a male partner [25–27], having a father with no formal education [25], alcohol consumption [24,25], substance/drug use [28], and not being able to freely discuss issues with their families [25].

In Ethiopia, political instability might contribute to young people’s exposure to violence, with universities often serving as focal points for its escalation. These institutions, characterized by diverse student populations and political discourse, create environments where tensions frequently lead to both the experience and initiation of violent acts. Previous studies of violence among university students in Ethiopia have only targeted one specific university and have focused solely on women/girls, often with an emphasis on gender-based violence [24–26,29]. Further knowledge is needed concerning the prevalence and factors associated with exposure to violence among both male and female university students. Therefore, this study aimed to determine the prevalence of exposure to violence and associated factors among male and female university students in Ethiopia. A better understanding of the dynamics of violence and the factors associated with exposure to violence among university students is necessary to improve prevention and intervention strategies in the university context.

## Methods

### Study design and setting

A cross-sectional study was conducted at six randomly selected public universities in Ethiopia: Hawasa, Dire Dawa, Bahir Dar, Ambo, Addis Ababa, and Adama University, located in different regions and city administrations. Bahir Dar and Hawassa Universities are located in scenic areas with cultural attractions, 552 km northwest and 278 km south of Addis Ababa, respectively. Dire Dawa and Adama Universities, about 515 km and 79 km east of Addis Ababa, are located in a region known for its industrial activities. Ambo University, 114 km west of Addis Ababa, is situated in an agricultural hub. Addis Ababa University is the oldest university, located in the capital city of Ethiopia.The data collection period was between August 2021 and February 2022.

### Study population

The study participants were undergraduate university students in their second and third year of study. First-year students were excluded since they were new to the university and their exposure to violence during their 12 months at the university may not have been fully established. Fourth- and fifth-year students were excluded because they had mostly completed their coursework and left the university campus for their apprenticeship.

### Sample size and sampling procedures

A single population proportion was used to determine the sample size. For each university, the calculated sample size was 493, considering a 45.4% prevalence of sexual violence among female university students [18], a 95% CI, a design effect of 1.2, and a non-response rate of 10%. The aggregated sample size for the six universities was 2958. However, all students from the selected departments who were willing to take part in the study were included, yielding a final sample of 2988.

To recruit study participants, a two-stage stratified sampling technique was used. First, universities were stratified into first- and second-generation universities to assess whether the risk of exposure to violence differed by the university’s year of establishment. First-generation universities (i.e., Addis Ababa, Bahir Dar, and Hawassa) are older institutions located in larger towns and have more established environments such as nightclubs and bars, compared to second-generation universities (i.e., Dire Dawa, Ambo, and Adama). Access to venues such as nightclubs and bars might increase the opportunities for the students to engage in risky behavior. During the study period, there were nine first-generation and twelve second-generation universities in Ethiopia, from each of which three universities were randomly selected using the lottery method.

Secondly, on average, 19 departments were randomly selected from each university. This was based on the assumption that there would be no difference between departments in terms of students’ likelihood of exposure to violence. Information about the lists of departments was obtained from the registrar of each university before data collection began. During class sessions, students were informed about the study and asked to participate voluntarily. Written consent was collected from those who agreed to participate, and they then completed a self-administered questionnaire in the classroom.

### Data collection tools

The questionnaire included background characteristics, alcohol and drug use, and exposure to emotional, physical, and sexual violence. The questionnaire was adapted from the Ethiopian Demographic and Health Survey questionnaire [30,31], the World Health Organization (WHO) alcohol consumption indicator book [32], and previous similar studies [17,24–26,33]. The questionnaire was developed in English and translated into Amharic and Oromifa by professional language translators. A pretest was done on 296 students (10% of the total sample size). Feedback from the pre-test results was incorporated into the final version of the questionnaire. Data collection coordinators were trained on data collection procedures, and the principal investigator closely monitored and supervised the data collection process.

### Variables: Definitions and assessment

#### Violence (dependent variable).

According to the World Health Organization (WHO), violence is the intentional use of physical force or power, threatened or actual, against oneself, another person, or against a group or community that either results in or has a high likelihood of resulting in injury, death, psychological harm, maldevelopment, or deprivation [34].

For this study, exposure to violence was assessed in terms of experience of emotional, physical, and sexual violence within the time frames “ever” and “during the last 12 months”. For the regression analyses, exposure to violence was defined as exposure to at least one type of violence at least once during the last 12 months. Emotional violence was assessed by the question, “Have you been exposed to any of the following threats or threats of violence (ever and in the past 12 months) that were so dangerous or serious that they scared you?” The response options were (Someone) “Said or did something to humiliate you in front of others”, “Threatened to hurt or harm you or someone close to you”, “Insulted you or made you feel bad about yourself”, “Other, specify” and “No”. Those who answered affirmatively to at least one of the options except “No” were considered to have experienced emotional violence. Physical violence was assessed by the question, “Have you been a victim of any of the following physical violence at any time (ever and during the past 12 months)?”, where the response choices were (Someone) “Pushed you, shook you, or threw something at you”, “Slapped you”, “Twisted your arm or pulled your hair”, “Punched you with his/her fist or with something that could hurt you”, “Kicked you, dragged you, or beat you up”, “Tried to choke you or burn you on purpose”, “Threatened or attacked you with a knife, gun, or any other weapon”, “Other, specify”, and “No”. Those who responded affirmatively to at least one of the options except “No” were considered to have experienced physical violence. Experience of sexual violence was assessed by the question “Have you ever been and during the past 12 months raped or forced to have sex against your will?” where the choices were “Yes” and “No”. Those who replied “Yes” were considered to have experienced sexual violence. Participants were also asked about the frequency of physical and sexual violence using an open-ended question, “In the past 12 months, how many times has someone physically hurt you/ raped you or forced you to have sex against your will?”. Regarding the perpetrator, information was obtained by the questions: “The last time this has happened, what was your relationship to the perpetrator? If it was more than one person, what was your relationship to the person who initiated the violence the most recent time this happened?”, where the response options were “Boyfriend/girlfriend”, “Teacher/lecturer”, “Other student/classmate”, “Husband/ wife”, “Family member”, “Person unknown to me/ stranger” and “Other (specify)”.

#### Sociodemographic characteristics (independent variables).

Information about age was obtained by an open-ended question that was grouped into three categories for the purpose of analysis: 18–20 years, 21–24 years, and older than 25 years. Relationship status was dichotomized as “in a relationship” and “not in a relationship.” “In a relationship” was defined as having a boyfriend or girlfriend, being married, or living together.

Residence was dichotomized as urban vs. rural, according to the Ethiopian Central Statistical Authority, which classifies a locality as urban residence if it has at least 2000 inhabitants, includes all administrative capitals of regions, zones, and woredas, or has at least 1000 people who are primarily engaged in non-agricultural activities and/or is declared urban by the administrative official. All areas not classified as urban were designated as rural residences [35].

Living status on campus was assessed by asking participants, “Where do you live?” The response options were “on campus” (living on university premises) and “off campus” (living outside the university premises). Participants were also asked about their living conditions during secondary school in the form of the question, “Did you mostly live at home or away from home while attending secondary school?” The response options were “at home” and “away from home.” Other demographic information collected included sex, religion, year of study, and faculty of study.

#### Alcohol consumption.

Regarding alcohol consumption, participants were asked whether they had ever experienced alcohol induced blackout, with the following response options: “I do not drink alcohol”, “I don’t remember”, “No”, I didn’t consume so much alcohol,” “Yes, before the past 12 months,” and “Yes, in the past 12 months.” Those who affirmed having this type of excessive consumption before the past 12 months and during the past 12 months are hereafter referred to as having excessive alcohol consumption and those who affirmed that they did not drink alcohol or did not consume excessive amounts of alcohol were considered as not having excessive alcohol consumption. Additionally, those who responded, “I don’t remember” were considered as missing. Participants were also asked about the frequency of their alcohol consumption in the past month, with the following response options: “I do not drink alcohol,” “Less often than once every two weeks,” “Once every two weeks or more,” “Once a week or more,” “Every day,” and “Other”. Those who drank every day and once a week or more were considered as drinking once a week or more. Those who drank once every two weeks or more and less often than once every two weeks were considered drinking less often than once a week. Furthermore, those who replied “other” without text were considered as missing. Participants were also asked about heavy episodic drinking (HED), defined as those who ever consumed five or more (for men) or four or more (for women) standard drinks of alcohol on at least one occasion in the past 30 days [36]. Thus, participants were asked, “Have you ever consumed four/five or more standard drinks of alcohol on at least one occasion?”, with the response options “Never”, “Yes, before the last 12 months” “Yes, in the last 12 months”, “Yes, in the last one month” and “Yes, in the last one week”. For the current analysis, those who responded “Yes, in the last one month” and “Yes, in the last one week” were considered as “Yes” (HED) and the rest of the options were considered as “No”.

#### Substance use.

Substance use was assessed by asking participants, “Have you used any substances/drugs/ intoxicants other than alcohol in the past 12 months?” The response options were “Khat”, “Ganja (Atsefaris)”, “Cocaine”, “Inhaling solvents such as benzine or glue”, “Marijuana (cannabis)”, “Never used”, and “Other, specify.” For the analysis, those who chose any of the options were categorized as “Yes”. Those who reported using substances other than khat, i.e., ganja (atsefaris), hashish, inhaling solvents, marijuana, and cannabis were considered as having “used any other drugs”. Khat (Catha edulis) is a flowering stimulant plant containing the alkaloid cathinone, which causes excitement and euphoria [37]. Ganja is a colloquial term used to refer to cannabis, particularly the dried flowers and leaves of the Cannabis sativa plant [38]. The frequency of substance or drug use was asked in the form of “How often have you used the drugs or intoxicants during the past month?” The response options were, “Less often than once every two weeks”, “Once a week or more”, “Once every two weeks or more”, “Every day”, “I do not use drugs or intoxicants”, and “Other specify”. Those who used drugs or intoxicants every day and once a week or more were considered as if they used once a week or more. In addition, those who used drugs or intoxicants once every two weeks or more and less often than once every two weeks were considered if they used less often than once a week. Furthermore, those who replied “other” without text were considered as missing. The frequency of substance or drug use includes all types of substances or drugs.

### Data analysis

The data were entered twice using EpiInfo version 7.2.2.12 by two independent data clerks in order to validate the consistency of the data and then exported to SPSS Version 26 for analysis. Data from all six universities were aggregated into a single dataset for the final analysis. Descriptive statistics, such as frequency and percentages, were used to summarize the data. Bivariable and multivariable logistic regression analyses were performed to identify factors associated with exposure to violence in the last 12 months. Crude and adjusted odds ratios with 95% confidence intervals (CIs) were used to measure the strength of the association. A p-value ≤  0.25 was used to select potential variables for the final multivariable analysis. A higher p-value was initially used to keep important variables in the model selection process [39]. A correlation analysis was also conducted to assess multicollinearity and no correlation was found between the variables. Associations with a p-value less than 0.05 were considered statistically significant. Cases with missing values for a particular variable were excluded from those analyses.

### Ethical considerations

Ethical approval was granted by the Scientific and Ethical Research Office (SERO) of the Ethiopian Public Health Institute (reference number EPHI 6.13/609). Written informed consent was obtained from each study participant prior to administering the questionnaire during their class sessions. The participants were informed that participation was voluntary and that they could withdraw at any point in the study process. No personally identifiable information was collected, and confidentiality of the information was maintained.

### Inclusivity in global research

Additional information regarding the ethical, cultural, and scientific considerations specific to inclusivity in global research is included in the Supporting Information (S1 Checklist).

## Results

### Socio-demographic characteristics of the participants

Table 1 presents the socio-demographic distribution of the study participants by gender.

**Table 1:** Socio-demographic characteristics by gender among undergraduate students at six universities in Ethiopia, 2022 (N =  2988).

Variable	Total n (%)	Male n (%)	Female n (%)
**Age** (n = 2758)			
18 - 20 years	770 (27.9)	395 (51.3)	375 (48.7)
21 - 24 years	1894 (68.7)	1330 (70.2)	564 (29.8)
>25 years	94 (3.4)	81 (86.2)	13 (13.8)
**Sex** (n = 2911)			
Female	1016 (34.9)		
Male	1895 (65.1)		
**Relationship status** (n = 2774)			
In relationship	542 (19.5)	305 (56.3)	237 (43.7)
Not in relationship	2232 (80.5)	1492 (66.8)	740 (33.2)
**Religion** (n = 2851)			
Orthodox Christian	1609 (56.4)	1011 (62.8)	598 (37.2)
Catholic	33 (1.2)	22 (66.7)	11 (33.3)
Protestant	731 (25.6)	492 (67.3)	239 (32.7)
Muslim	433 (15.2)	290 (67.0)	143 (33.0)
Other	45 (1.6)	37 (82.2)	8 (17.8)
**Residence before coming to the university** (n = 2794)			
Urban	2044 (73.2)	1272 (62.2)	772 (37.8)
Rural	750 (26.8)	552 (73.6)	198 (26.4)
**Living conditions while attending secondary school** (n = 2829)			
At home	2188 (77.3)	1347 (61.6)	841 (38.4)
Away from home	641 (22.7)	493 (76.9)	148 (23.1)
**Generation of the university** (n = 2911)			
First generation	1467 (50.4)	978 (66.7)	489 (33.3)
Second generation	1444 (49.6)	917 (63.5)	527 (36.5)
**Faculty** (n = 2890)			
Faculty of Natural and Computational Science	521 (18.0)	351 (67.4)	170 (32.6)
Faculty of Medicine	48 (1.7)	30 (62.5)	18 (37.5)
Faculty of Social and Human Science	619 (21.4)	367 (59.3)	252 (40.7)
Faculty of Law	224 (7.7)	136 (60.7)	88 (39.3)
Faculty of Business and Economics	751 (26.0)	491 (65.4)	260 (34.6)
Faculty of Institute of Technology	727 (25.2)	510 (70.2)	217 (29.8)
**Year of study** (n = 2911)			
Second-year student	1424 (48.9)	877 (61.6)	547 (38.4)
Third-year student	1487 (51.1)	1018 (68.5)	469 (31.5)
**Living status on campus** (n = 2777)			
On campus	2578 (92.8)	1726 (67.0)	852 (33.0)
Off-campus	199 (7.2)	92 (46.2)	107 (53.8)

Out of 3165 students in selected departments, 2988 participated in the study. The majority of participants (65.1%) were male. The largest proportion of students were aged between 21-24 years (68.7%), 80.5% were not in relationships, more than half (56.5%) were Orthodox Christian, and about three-fourths (73.3%) were from urban residences. In addition, about half of the participants (50.4%) were from first-generation universities and about one-fourth (26.0%) were from the Faculty of Business and Economics. The majority (92.8%) of the students were living on campus and half (48.9%) of them were in their second year of studies (Table 1).

Table 2 shows the distribution of alcohol consumption and substance use among the study participants. Regarding alcohol consumption, 9.3% of the students had ever had alcohol induced blackout. Only 4.8% of participants reported their alcohol consumption as weekly or more often in the past month. Of the study participants, 5.9% were classified as having had HED in the past month.

**Table 2:** Alcohol consumption and substance use by gender among undergraduate students at six universities in Ethiopia, 2022 (N =  2988).

Variable	Total n (%)	Male n (%)	Female n (%)
**Alcohol induced blackout** (n = 2624)			
Yes	245 (9.3)	172 (70.2)	73 (29.8)
No	2379 (90.7)	1549 (65.1)	830 (34.9)
**Frequency of alcohol drinking in the last month** (n = 2477)			
Once a week or more	120 (4.8)	87 (72.5)	33 (27.5)
Less often than once a week	344 (13.9)	256 (74.4)	88 (25.6)
Did not drink alcohol	2013 (81.3)	1279 (63.5)	734 (36.5)
**Heavy episodic drinking in the last month** (n = 2740)			
Yes	161 (5.9)	112 (69.6%)	49 (30.4)
No	2579 (94.1)	1665 (64.6)	914 (35.4)
**Ever chewing khat** (n = 2654)			
Yes	286 (10.8)	224 (78.3)	62 (21.7)
No	2368 (89.2)	1514 (63.9)	854 (36.1)
**Chewing khat in the last 12 months** (n = 2794)			
Yes	195 (7.0)	152 (77.9)	43 (22.1)
No	2599 (93.0)	1668 (64.2)	931 (35.8)
**Ever used any other drugs** (n = 2654)			
Yes	230 (8.7)	157 (68.3)	73 (31.7)
No	2424 (91.3)	1581 (65.2)	843 (34.8)
**Used any other drugs in the last 12 months** (n = 2795)			
Yes	189 (6.8)	126 (66.7)	63 (33.3)
No	2606 (93.2)	1695 (65.0)	911 (35.0)
**Frequency of all types of substance use in the last month** (n = 2703)			
Once a week or more	140 (5.2)	111 (79.3)	29 (20.7)
Less often than once a week	166 (6.1)	122 (73.5)	44 (26.5)
Did not use substance or drug	2397 (88.7)	1534 (64.0)	863 (36.0)

Concerning substance/drug/intoxicant use, 10.8% of respondents had ever used khat and 7.0% of them used khat in the last 12 months. Further, 8.7% and 6.8% of them used other drugs ever and in the last 12 months, respectively. Regarding the frequency of drug use in the past month, 5.2% of them used once a week or more. The majority of substance/drug/intoxicant users were men as shown in Table 2.

### Exposure to violence

The prevalence of exposure to any type of violence ever was 24.9% (n = 742), while the prevalence of exposure to violence in the last 12 months was 17.6% (n = 525). The prevalence of exposure to emotional, physical and sexual violence in the last 12 months was 17.8% (n = 508), 12.3% (n = 349) and 3.3% (n = 87), respectively.

**Fig 1:**
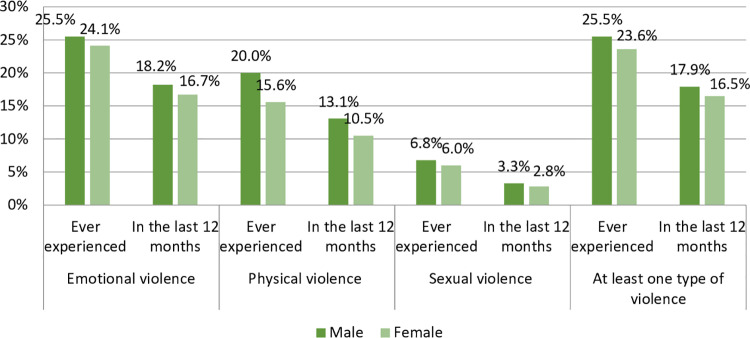
Exposure to different types of violence by gender, 2022.

The prevalence of exposure to at least one type of violence ever among males and females was 25.5% (n = 482) and 23.6% (n = 240), respectively, whereas the prevalence of exposure to violence among males and females in the last 12 months was 17.9% (n = 340) and 16.5% (n = 168), respectively (Fig 1).

Regarding the frequency of violence experience, 41.9% of the students experienced physical violence more than once and 13% of the students experienced sexual violence more than once.

Table 3 shows the proportion of students who experienced different forms of violence and the type of perpetrator who had initiated the violence on the most recent occasion.

**Table 3:** Perpetrators and different types of violence experienced by undergraduate university students in Ethiopia, stratified by gender of respondent, 2022.

Perpetrators	Emotional violence (n = 623)				Physical violence (n = 399)				Sexual violence (n = 143)			
	Male		Female		Male		Female		Male		Female	
	N	%	N	%	N	%	N	%	N	%	N	%
Boyfriend/girlfriend	130	31.3	41	19.7	73	25.9	28	23.9	40	43.0	17	34.0
Teacher/lecturer	65	15.7	34	16.3	42	14.9	13	11.1	22	23.7	7	14.0
Other student/ classmate	102	24.6	42	20.2	82	29.1	23	19.7	8	8.6	11	22.0
Husband/wife	23	5.5	16	7.7	5	1.8	3	2.6	2	2.2	3	6.0
Family member	33	7.9	26	12.5	28	9.9	18	15.4	11	11.8	8	16.0
Person unknown to me/ stranger	40	9.6	34	16.3	36	12.8	25	21.4	3	3.2	3	6.0
Other	22	5.3	15	7.2	16	5.7	7	6.0	7	7.5	1	2.0

Note: Cases with missing data were not included in the analysis

Intimate partners (boyfriends/girlfriends) were the most common perpetrators of emotional violence (27.7%) and sexual violence (39.3%). With regard to physical violence, other students/classmates were the most frequent perpetrators (25.5%).

### Factors associated with violence as shown by bivariable and multivariable logistic regression analysis

Table 4 shows the results of the bivariable and multivariable logistic regression analyses for factors contributing to violence in the last 12 months.

**Table 4:** Bivariable and multivariable logistic regression analysis of factors associated with exposure to violence among undergraduate university students in Ethiopia, 2022 (N =  2988).

Variables	COR (95% CI)	p-value	AOR (95% CI)	p-value
**Age**				
18 - 20 years	1		1	
21 - 24 years	1.2 (1.0-1.5)	0.119^*^	1.1 (0.8-1.5)	0.422
>25 years	2.9 (1.8-4.6)	<0.001^*^	2.9 (1.6-5.0)	<0.001^**^
**Sex**				
Female	0.9 (0.7-1.1)	0.341		
Male	1			
**Relationship status**				
In relationship	1.6 (1.3-2.0)	<0.001^*^	1.4 (1.0-2.0)	0.002^**^
Not in relationship	1		1	
**Religion**				
Orthodox Christian	1		1	
Catholic	2.4 (1.1-4.9)	0.022^*^	1.9 (0.7-5.6)	0. 228
Protestant	1.0 (0.8-1.3)	0.987	0.9 (0.7-1.2)	0.528
Muslim	1.0 (0.7-1.3)	0.814	1.0 (0.7-1.5)	0.813
Other	1.0 (0.5-2.1)	0.940	1.1 (0.4-2.8)	0.844
**Residence before coming to the university**				
Rural	1.4 (1.1-1.7)	0.003^*^	1.4 (1.1-1.8)	0.015^**^
Urban	1		1	
**Generation of the university**				
First	1.3 (1.0-1.5)	0.016^*^	1.4 (1.1-1.8)	0.006^**^
Second	1		1	
**Year of study**				
Second-year student	1		1	
Third-year student	1.2 (1.0-1.4)	0.156^*^	1.0 (0.8-1.3)	0.821
**Living status on campus**				
In campus	1		1	
Out of campus	0.7 (0.5-1.1)	0.081^*^	0.6 (0.4-1.1)	0.111
**Frequency of alcohol drinking in the last month**				
Once a week or more	2.8 (1.9-4.2)	<0.001^*^	2.2 (1.3-3.6)	0.002^**^
Less often than once a week	1.8 (1.3-2.3)	<0.001^*^	1.4 (1.0-2.1)	0.050
Did not drink alcohol	1		1	
**Heavy episodic drinking in the past one month**				
Yes	2.0 (1.4-2.8)	<0.001^*^	1.0 (0.6-1.7)	0.975
No	1		1	
**Chewing khat in the last 12 months**				
Yes	1.8 (1.3-2.5)	<0.001^*^	1.6 (1.0-2.4)	0.039^**^
No	1		1	
**Used any other drugs in the last 12 months**				
Yes	2.9 (2.2-4.0)	<0.001^*^	2.0 (1.3-3.1)	0.001^**^
No	1		1	

* P-value < 0.25;

** P-value <  0.05; COR =  Crude Odds Ratio; AOR =  Adjusted Odds Ratio; CI = Confidence interval; 1 = Reference category.

Note: P-value < 0.25 was used as the cut-off point for inclusion in the multivariable analysis.

The bivariable regression analysis showed that being older than 25 years, being in a relationship, being from a rural residence before coming to the university, studying at first-generation universities, drinking alcohol once a week or more and less often than once a week in the past month, having HED in the past month, chewing khat and using other drugs in the last 12 months were significantly associated with violence (p-value < 0.05). In addition to the aforementioned variables, the variables aged between 21-24 years, being Orthodox Christian, being a third-year student, and living off-campus were selected for multivariable regression analysis (p-value < 0.25) (Table 4).

In multivariable logistic regression analysis, when all variables were mutually adjusted for one another, the adjusted odds of experience of violence were 2.9 times higher (95% CI 1.6-5.0) among students older than 25 years compared to those aged 18-20 years. Students who were in a relationship had 1.4 times higher likelihood of violence (95% CI 1.0-2.0) than those who were not in a relationship. In addition, students who were from rural residences before coming to the university had 1.4 times higher odds of violence (95% CI 1.1-1.8) as compared to those from urban residences. Students studying at first-generation universities had 1.4 times higher (95% CI 1.1-1.8) odds of violence than students at second-generation universities. The odds of violence were 2.2 times higher (95% CI 1.3-3.6) among those who consumed alcohol once a week or more compared to those who did not drink alcohol in the past month. Furthermore, the odds of violence were 1.6 times higher (95% CI 1.0-2.4) among those who had chewed khat and 2 times higher (95% CI 1.3-3.1) among those who used other drugs (Table 4).

## Discussion

To our knowledge, this is the first study that examines exposure to violence both among male and female university students in Ethiopia and presents data from six universities. The overall prevalence of exposure to at least one type of violence (emotional or/and physical or/and sexual violence) ever was 24.9%, whereas the prevalence in the last 12 months was 17.6%, with similar exposure prevalence among males and females, 17.9% and 16.5%, respectively. The odds of exposure to violence in the last 12 months were higher among students older than 25 years, those who were in a relationship, those with rural residence before coming to the university, those who consumed alcohol more frequently, and those who used khat and other drugs in the last 12 months.

The current study shows that exposure to any form of violence was common among both male and female university students. This might be due to university students experiencing difficulties in adjusting to a new environment, managing newfound independence and experimenting with different risky lifestyles such as excessive consumption of alcohol and substance use. Comparing the prevalence of exposure to violence across studies can be difficult due to various types of methodological differences, including sampling and definitions of violence. Furthermore, previous studies in Ethiopia have largely concentrated on gender-based violence, primarily focusing on women and girls, thus making it difficult to compare the prevalence of violence among males and females in the current study with any other locally conducted studies. Nevertheless, the prevalence of violence among males and females in the last 12 months in this study was similar to the prevalence obtained in a study conducted among university students in the US and Canada, showing 17% and 16% among males and females, respectively [14]. Even though their setting is different from our study, the study was similar in terms of study design, study population, and age. The overall prevalence of experience of violence at two universities in Mexico was 25.7% and 31.9% [40] which is higher than the current study, most likely because violence was measured as the sum of four types of violence (verbal, psychological, sexual, and physical violence), whereas in our study, those who experienced at least one type of violence were considered as having violence exposure.

In this study, the prevalence of emotional violence (17.8%) was lower, while the prevalence of physical violence (12.3%) in the last 12 months was higher compared to results from a study conducted among university students in southwestern Uganda, which found a prevalence of 27.8% and 9.6%, respectively [16]. This might be due to methodological differences. The study in Uganda included the entire undergraduate class of the university and used a different formulation with regard to the violence questions. Our study used questions with different categorical options, whereas the Ugandan study used response options in the form of “yes” or “no”. Furthermore, a study on violence against dating partners by male and female university students worldwide showed that 7% had been physically injured by a partner (range =  2% to 20%) in the past 12 months [41]. The finding of the current study falls within this range. The experience of physical violence within a relationship could be due to the power imbalance, jealousy or possessiveness in a relationship, as shown in a studies of jealousy and intimate partner violence among university students in Ecuador [42] and young adults in the US [43].

Unexpectedly, the prevalence of sexual violence was higher among males than females in the current study. This might be due to the fact that sexual violence is a very sensitive issue in Ethiopia, where strict social norms and cultural factors play a significant role. Thus, female students may underreport incidents of sexual violence due to stigma, fear of not being believed, or concern about potential consequences. According to the EDHS, among women aged 15 to 49 years who experienced either physical or sexual violence or both, 66% have never disclosed their exposure to anyone [6]. Similarly, a systematic review and meta-analysis of female domestic violence disclosure in Ethiopia showed a pooled prevalence of 36.2% [44]. The reasons for not disclosing were considering violence as normal or not serious, shame, embarrassment, and fear of disclosure related consequences [44]. An internet-based study of undergraduate females in New York City and Miami, Florida showed that among participants who reported sexual victimization, 25% had not previously disclosed it because of shame, minimization of experience, fear of consequences, and privacy [45]. Furthermore, a study among adult female sexual assault survivors in the United States revealed that the reasons for not telling people were fear of negative social reactions, lack of perceived available support, fear of burdening others, and family and social norms expectations [46]. In this study, the prevalence of sexual violence among females was lower compared to findings from a study conducted among female university students in Wolaita Sodo [18], and a systematic and meta-analysis conducted among female university students in Ethiopia [47,48]. This could be due to the difference in the operational definition of sexual violence, as the other studies included attempted rape, and also variations in study design, with the systematic review and meta-analysis encompassing all types of observational studies. The ecological model can help us understand the importance of exploring how factors such gender and gender norms could affect vulnarabilities to, and behaviors associated with, violence [49,50]. Our study showed no significant gender differences in overall exposure to violence, which is similar to a study among university students in southwestern Uganda [16]. However, a study conducted among university students in Italy showed that ever experienced psychological and physical violence among peers/school was reported significantly more often by males than by females (21.5% vs. 9.7%) [15]. Another study among university students in Finland showed that men reported more emotional and physical violence than women [51], which is similar to the current study. This might be due to the societal norms and expectations around masculinity that encourage confrontation among men [52,53]. In addition, the current findings of a higher prevalence of emotional violence among male students vs. female students could be due to male students in Ethiopia engaging in risky behaviors such as alcohol consumption [7,54,55] and drug use [10,12,56].

In the microsystem described by Bronfenbrenner’s immediate relationships with family, peers and teachers can play a role in contributing to violent behaviors [20]. In our study, most of the perpetrators of emotional violence were boyfriends/girlfriends, which is in line with a study among university students in the US and Canada [14] which found that nearly half of the students experienced violence from intimate partners or persons whom the students were dating or with whom they were in an ongoing romantic relationship. However, physical violence perpetrators were other students/classmates and also boyfriends/girlfriends, albeit to a lesser extent, which is similar to the aforementioned study [14]. Furthermore, the perpetrators of sexual violence were boyfriends/girlfriends and also teachers/lecturers, albeit to a lesser extent, which is in line with a study conducted on sexual violence among female university students in Ethiopia [57]. The patterns of perpetrator characteristics shown here could be linked to experiences of childhood abuse and growing up with domestic violence, factors which have previously been shown to be significantly associated with intimate partner violence in a multi-country population-based study among women [58]. The relevance of childhood abuse experiences would also be suggested by Bronfenbrenner’s model where children who experience violence at home may develop unhealthy coping mechanisms and encounter difficulties in establishing and maintaining healthy relationships [19]. Similarly, the authority held by lecturers or teachers can lead to situations where students may be vulnerable to manipulation or abuse [59,60].

Another individual factor contributing to violence exposure is age. In the current study, students who were older than 25 years had higher odds of having experienced violence in the last 12 months. This finding is in line with a study on exposure to emotional violence among university students in Turkey [23], which showed that the level of exposure to emotional violence increased as age increased. Another study on the effect of sex and age on experiences of violence during the past year among adolescents aged 13 to 24 years in five countries (Cambodia, Haiti, Kenya, Malawi, and Tanzania) [61] revealed that the risk of sexual violence and also intimate partner violence increased with age. This could be due to older students being more prone to alcohol or substance use than their younger counterparts [62–64], which can impair their judgment and elevate the likelihood of encountering violent situations. A study conducted on the magnitude of and trends in alcohol-related mortality and morbidity among U.S. college students revealed that excessive alcohol consumption increased with age and alcohol consumption was associated with violence experience among the students [65].

Bronfenbrenner’s ecological model suggests that immediate social environments such as family dynamics, peer influences, and intimate relationships play a crucial role in shaping an individual’s behavior [20,21]. These could significantly affect the likelihood of experiencing or perpetrating violence where negative interactions in a relationship can create a cycle of conflict and aggression, reinforcing patterns of violent behavior over time. In the current analysis, students who were in a relationship had higher odds of violence. Romantic partners could spend a long time together, which can create opportunities for conflicts and disagreements that can escalate into violence. Additionally, dependency in romantic relationships can make individuals more vulnerable to manipulation and abuse. Being in a relationship was previously found to be significantly associated with sexual violence among female university students in Ethiopia [18,57]. This factor was also significantly associated with gender-based violence (GBV) among female students in Ethiopia [25,26], at public universities in South Africa [27] and among male and female university students in the US and Canada [14]. Being in a relationship would de facto increase the risk of experiencing intimate partner violence, including emotional, physical, or sexual violence by a partner.

Another factor significantly associated with violence was having a rural residence prior to attending the university. This is similar to a study conducted on sexual violence among female students at Bahir Dar private college [66] in Ethiopia, which revealed that rural residence was significantly associated with exposure to different types of violence in the academic setting. This could be due to students from rural residences being exposed to forms of violence such as teasing and bullying, potentially because they are perceived as different or outsiders. A study conducted on structural family factors and bullying at school among adolescents in China showed that being from a rural residence was associated with bullying at school [67]. Furthermore, the influence of rural residence is supported by Bronfenbrenner’s ecological model, suggesting that broader social systems indirectly influence an individual’s development, such as the community and neighborhood in which they live [68].

In the absence of parental oversight, students may feel a newfound sense of freedom and independence, which could result in experimenting with risky behaviors such as drinking alcohol and substance use. Additionally, the influence of peers plays a significant role; students may be encouraged or feel compelled to partake in these behaviors to fit in or gain acceptance within their social circles. Students often engage in these risky behaviors in social settings, such as parties or gatherings, where the dynamics of the group can intensify these actions. The collective influence of peers can amplify tendencies toward excessive drinking or drug use [69–71]. This is also in line with Bronfenbrenner’s ecological model where peer pressure can influence individuals’ behaviors [20,21]. Such an environment can also heighten aggressive behavior and consequently, this can escalate into violent incidents.

In the current study, the odds of violence were higher among those who consumed alcohol more frequently. The effects of alcohol include a reduction in cognitive and physical functions that impair self-control, with the consequent effect of reducing the ability to resolve conflicts peacefully [72]. Studies in Ethiopia on sexual violence and GBV among female college/university students [24,25,18] revealed that those students who habitually drank alcohol were more likely to be victims of violence. A research review of alcohol consumption among college students showed that those students who binge drink frequently (three or more times in two weeks) were at particularly high risk of negative alcohol-related outcomes [73]. Being a college or university student may involve a special risk for young adults, as increased availability of alcohol and acceptance of drinking on college campuses may lead to increases in unwanted consequences, including physical and sexual assaults [74].

Furthermore, in the current study, those who used khat and other drugs had higher odds of exposure to violence. Studies conducted among female college and university students in Ethiopia showed that khat was significantly associated with GBV [17,24,57]. A study on violence among college students in India found a significant association between substance use and violence [28]. In addition, physical violence was significantly associated with illicit drug use in the past 30 days in a study conducted in the US [75]. Moreover, consumption of alcohol or other drugs was significantly associated with experiences of harassment among students in a study in Spain [76]. Both alcohol and substance use can impair cognitive function and decision-making abilities, lower inhibitions, and increase aggression, which potentially leads to greater exposure to violence. Furthermore, there is a complex causal relationship between violence and alcohol and drug use, where these substances are often used as coping mechanisms after traumatic events, such as experiences of violence [77,78]. Additionally, being in environments like bars can increase vulnerability, as the presence of intoxicated individuals can heighten the likelihood of violence, even if one does not drink to the extent of losing judgment. Therefore, universities could create a comprehensive approach to reduce violence related to risky behaviors, such as implementing comprehensive education programs about the risks and consequences of alcohol and drug use, offering accessible counseling services that provide support for students struggling with substance use [79,80], and working with local businesses to limit the availability of alcohol and substances near campus [81,82]. A comprehensive approach would be in line with Bronfenbrenner’s ecological model, indicating the need to address multiple contributing factors across individual, relational, community, and societal levels that contribute to violence. This holistic perspective could inform the development of effective prevention and intervention strategies tailored to university students.

### Methodological considerations

While this study is a comprehensive exploration of violence among university students in Ethiopia, the findings might be specific to the studied population, and caution is needed when generalizing to university students broadly or students in other countries because of differences in culture and socioeconomic background.

Self-administration of the questionnaire may have led to incomplete, inaccurate, underreported, or overreported data. To mitigate these issues, participants were provided with orientation on how to complete the questionnaire before it was administered. Social desirability bias may have been present due to the sensitive nature of some questions, but the anonymity of study participation, where students placed their completed questionnaires in a designated box and the use of external data collection coordinators helped to reduce this source of bias. This study examines violence exposure among both male and female participants but does not assess the gender of the perpetrators. Although there were some missing data points in the responses to the questionnaire, no systematic pattern was observed, and the distribution of missing data was considered to be random.

## Conclusion

Exposure to violence is a prevalent challenge among female and male university students in Ethiopia. Socio-demographic and behavioral factors such as the use of alcohol and substances were significantly associated with exposure to violence. Universities, together with stakeholders such as the Ministry of Science and Higher Education, Ministry of Health, students, and partners that work on violence need to strengthen/develop awareness raising activities targeting violence prevention mechanisms and its associated factors, regardless of gender.

## Supporting information

S1 File Checklist. (PDF)
